# Sickle cell disease screening: Experiences of parents with positive newborns at Korle Bu TeachingHospital

**DOI:** 10.1371/journal.pone.0336204

**Published:** 2026-10-15

**Authors:** Irene Kanyoke, Faustina Hayford Blankson, Diana Dwuma-Badu, Catherine Segbefia

**Affiliations:** 1 Department of Social and Behavioral Sciences, School of Public Health, University of Ghana, Legon, Ghana; 2 Department of Child Health, Korle Bu Teaching Hospital, Accra, Ghana; 3 Department of Child Health, Korle Bu Teaching Hospital, Accra, Ghana; 4 Department of Child Health, University of Ghana Medical School, Accra, Ghana; Mulago National Referral Hospital, UGANDA

## Abstract

**Objective:**

To explore parents experiences following receipt of positive newborn screening for sickle cell disease (SCD) test results at a teaching hospital in Ghana.

**Methods:**

Using purposive sampling, data were collected using semi-structured individual guides to interview parents of children attending the Paediatric SCD clinic at Korle Bu Teaching Hospital (KBTH), a major referral health facility in Ghana. Questions were categorized into 3 main areas: parents’ reactions to initial positive results, parents’ responses to in-clinic counselling, and parents’ attitudes towards follow-up clinic care. The Socio-Ecological Model of Health was used to underpin this study. Interviews were conducted face-to-face either in English or a local dialect (Twi), after which they were transcribed verbatim. Data were analyzed using NVivo software and Microsoft Word to identify relevant codes, which were then organized into themes and sub-themes.

**Results:**

Twenty-four mothers and one father were interviewed between 2nd November 2021 and 28th January 2022. The ages of their children ranged from 7 months to 3 years 9 months. Seventeen (68%) of the parents were married, 5 (20%) had more than one child enrolled in the clinic, and 10 (40%) reported that they or their partners had SCD. The 7 themes identified were: (i) emotional response after disclosure, (ii) decision to share results, (iii) care and management of newborns at home, (iv) knowledge of genetics of SCD, (v) adherence to routine follow-up, (vi) cost, and (vii) Culture. Positive test results elicited worry, surprise, or shock in the majority, especially those with no previous knowledge of or experience with SCD. Most parents had received counselling and educational information on caring for their positive SCD children at home and the danger signs of SCD. The decision to share test results with others was influenced by fear of stigmatization. While desiring to see their children remain healthy, parents defaulted on clinic visits due to financial difficulties and the COVID-19 pandemic.

**Conclusion:**

Knowledge of SCD and personal experience influenced parents’ reaction to initial positive newborn screening for SCD test results. Culture and Cost were structural barriers to care. There is a need for more education about SCD among the general population and institutions, and there is a need to provide psychological and socioeconomic support for families attending the KBTH clinic.

## Introduction

Sickle Cell Disease (SCD) is a hereditary disorder characterized by the presence of abnormal haemoglobin, the most common of which is haemoglobin S (Hb S) [1–3]. SCD clinically presents as acute haemolytic and vaso-occlusive crises, which can lead to complications including stroke, organ damage, priapism and acute chest syndrome, among others [4–6], and it is estimated that between 300,000 and 500,000 babies are born with SCD worldwide each year [4,7]. Studies show that 7 in 10 SCD-related deaths are preventable, and the high prevalence of morbidity and mortality in Africa is made worse by inadequate systemic screening for the disease, inadequate follow-up care, shortage of facilities for SCD management, and poor research and intervention protocols [8,9].

SCD has a huge socio-economic impact not only on parents but also on the community and even the country at large [8,10], and as such, over the years, there have been several disease management interventions for the disease. One of these interventions is Newborn Screening (NBS). Newborn screening for SCD is the process by which blood from a baby is collected in the first few days after birth and tested for SCD [11,12]. The main objective of newborn screening is to allow for the early identification of infants with the disease and the provision of follow-up care to help reduce morbidity and mortality [13–15]. Newborn screening for SCD is a laudable idea, and comprehensive follow-up care, which involves a multi-disciplinary approach, must include the families and caregivers of newborns with SCD [14,16,17].

In Ghana, universal newborn screening, however, is not fully implemented [18], and in the few places where screening is taking place, barriers to care include parents’ refusal to accept an initial positive diagnosis or failing to attend a clinic after the initial visit [19]. In Ghana, the Clinton Health Initiative (CHAI), with funding from the Bill and Melinda Gates Foundation, expanded screening to all the then 10 regions of Ghana in 2021 and by 2023, over 24, 000 babies had been screened, with 397 testing positive for presumptive SCD (P-SCD) [20]. However, barriers to care meant that only 204 babies (51%) were linked to care [20]. In Korle Bu Teaching Hospital, Ghana, where newborn screening for SCD started with funding from Pfizer in 2017, the initial linkage to care was successful, with 74 (94%) babies of the initial 79 PSCD contacted after a positive diagnosis and 60 of those (79%) linked to the hospital’s Paediatric Sickle Cell Clinic (PSCC) for comprehensive follow-up care [14,21]. However, in the subsequent year, with an increased screening coverage, they were able to contact 121 (92%) of positive cases but only 62 of those (47%) accepted the initial results and were enrolled into a clinic for care [14,21]. In Nigeria, a study to test the feasibility of newborn screening with early linkage to comprehensive follow-up care (less than 3 months) found that less than half of infants who tested positive were enrolled into a clinic for follow-up care before age 3 months and that enrollment only increased following a targeted and enhanced family-centered communication strategy [22]. There is therefore an obvious disconnect between screening and linkage to care. This disconnect defeats the fundamental principle of newborn screening: enrollment into a clinic for comprehensive follow-up specialist care, including starting penicillin prophylaxis by age 3 months.

Due to these aforementioned deficiencies, this study aimed to explore the experiences of parents of children following the receipt of positive NBS results for SCD at a teaching hospital in Ghana.

### Theoretical framework

This study used an adaptation of Urie Bronfenbrenner’s Social-Ecological Model (SEM) developed in 1979 to underpin the study (Refer to Fig 1). The SEM has been adopted and adapted by public health experts over the years for research into understanding issues affecting well-being. Thus, this framework was adapted for this study to better explore the multifaceted factors that affect parents’ experiences.

**Fig 1:**
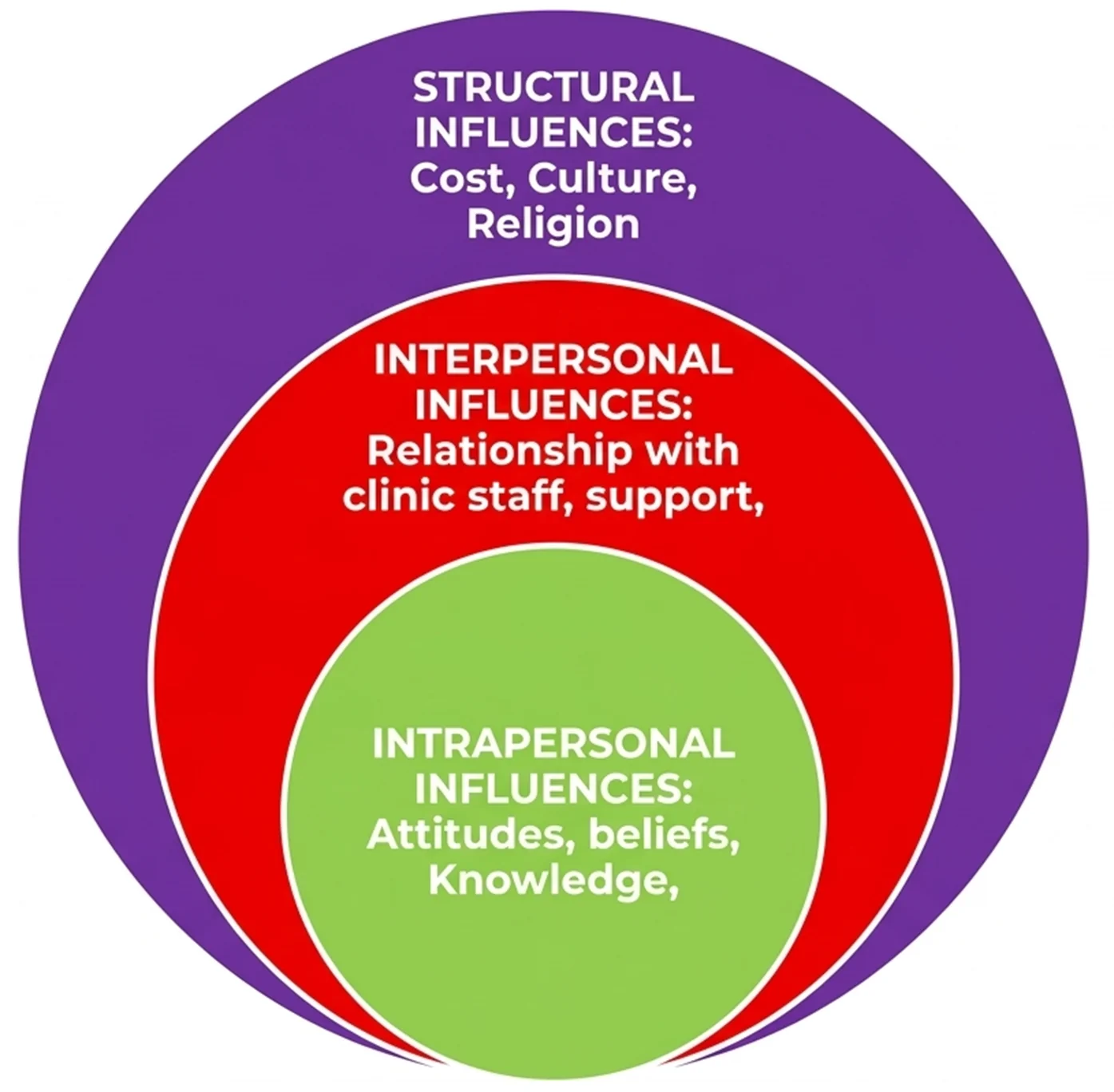
Illustration of the Socio-Ecological Model Adapted from Bronfenbrenner (1979).

### Social-ecological model of health

The SEM framework has been used by various health experts to gain an understanding of how various factors interplay to affect health and thus is useful in health prevention, control and promotion. The SEM has 5 levels of influence on health behaviour. This study, however, used an adapted version that examined the interplay and interconnectedness of individual (intrapersonal) factors such as knowledge and attitudes, interpersonal factors such as counselling at clinic and relationship with clinic staff, as well as structural factors such as cost and culture to critically assess the experiences of parents of positive SCD children at the Paediatric Sickle Cell Clinic (PSCC) of Korle Bu Teaching Hospital. The Social Ecological Model (SEM) was suitable as a framework for this study because it provided a robust platform to explore the complexities of individual factors such as parents’ reaction to positive results and their attitudes to follow-up care, as well as their networking or relationship with clinic staff, which helped them gain knowledge on how to care for their children with sickle cell disease and the genetics of the disease. It also helped the researcher explore how the interaction between these factors could influence early enrollment and encourage adherence to follow-up care at the clinic.

### Intrapersonal influence

The intrapersonal level of the model looks at the individual’s characteristics such as knowledge, skills, emotions, age, income, etc. This stage of the model looks at the individual at the centre of the theory and examines how certain characteristics of the individual influence certain health behaviors. The researcher, therefore, examined how micro and macro-level factors played a role in parents’ reactions to positive results, their responses to counselling in clinic and the attitudes of parents to follow-up clinic procedures. An individual’s knowledge, attitudes and feelings have a direct effect on their experiences and health-seeking behaviour. The SEM helped assess how some of these factors at the individual level influenced parents’ initial reaction to disclosure of positive results, their responses to counselling and information given at clinic and their behaviour towards adherence. This study adapted this model to better explore the role of individual influences in the experiences of parents of newborns who tested positive for SCD at the PSCC of Korle Bu Teaching Hospital.

### Interpersonal influence

Interpersonal factors such as relationships, interactions and interconnectedness that an individual has with other people, such as family, friends, or a health worker, influence health behaviour. It looks at the network of interactions between an individual and other people and how this interaction influences health. The SEM was used to examine the complexities of the relationships and interactions between clinic staff and parents. It explored how the interactions in clinic explain parents’ responses to in-clinic counselling and education about sickle cell disease. Framing the impact of educational counselling under this framework helped examine parents’ knowledge of the genetics and complications of SCD and management of the disease.

### Structural influence

Macro-level factors such as community, culture (religious beliefs), access to health care, and cost influence parents’ reaction to positive SCD results of their children, their understanding of the disease, as well as their adherence to follow-up clinical procedures. Individuals exist in societies, and examining how they interact within the structures of society helps examine their influences on health behaviour. Parents’ acceptance of positive results was influenced by their religious beliefs and societal perceptions of the disease.

### Research questions

The research questions that guided the study were:

**Research question 1 (RQ1):** What are the reactions of parents towards the communication of positive results?
**Research question 2 (RQ2)**: What are the responses of parents of positive SCD newborns towards in-clinic counselling?
**Research question 3 (RQ3):** What are the attitudes of parents of SCD newborns towards follow-up clinic procedures?

## Main objectives

The main objective of this study was to explore the experiences of parents of children with positive sickle cell disease results at the Paediatric Sickle Cell Clinic (PSCC) of Korle Bu Teaching Hospital (KBTH).

### Specific objectives

The specific objectives were:

1. To explore the reactions of parents of newborns towards disclosure of positive SCD results at the PSCC of KBTH.
2. To explore the responses of parents of newborns with P-SCD to in-clinic counselling and support at the PSCC of KBTH.
3. To describe the attitudes of parents of newborns with SCD towards subsequent follow-up clinic care at the PSCC of KBTH.

## Method

### Study design

This study used a qualitative research design with an exploratory approach, with data collection done using a semi-structured interview guide to conduct individual in-depth interviews.

### Study site

The study was conducted at the Paediatric Sickle Cell Clinic of the Korle Bu Teaching Hospital (KBTH), and the study population consisted of mothers or fathers of children enrolled in the Paediatric Sickle Cell Clinic of KBTH.

### Study participants

The study population were mothers or fathers of newborns enrolled in the Paediatric Sickle Cell Clinic of KBTH.

### Inclusion criteria

Inclusion criteria were all mothers or fathers of newborns diagnosed via the KBTH NBS program and enrolled in the clinic.

### Exclusion criteria

The exclusion criteria were parents of children diagnosed via the KBTH NBS programme and enrolled in the clinic, but attending the clinic for the first time or whose children were less than 6 weeks old.

### Sampling method and size

The researchers used the purposive sampling method to recruit participants. According to Francis et al, data saturation for theory-based qualitative studies can be decided by using 2 principles: 1. establishing an initial minimum sample size for analysis and 2. specifying the stopping criterion, i.e., how many more interviews will be conducted without new ideas [23]. A total of eighteen [18] interviews were set as the minimum sample size, with the stopping criterion being three [3]. New themes (emotional responses) stopped emerging by the 21^st^ interview. The researcher used 2 interview guides: a questionnaire to collect socio-demographic information and a semi-structured individual interview guide.

### Recruitment of participants

Participants were recruited from the Paediatric Sickle Cell Clinic of KBTH. They were mothers and one father who met the inclusion criteria. In one case, both parents of a child were available in the clinic on a particular day; as such, they were both interviewed together. Participants were recruited at the clinic using purposive and convenience sampling methods.

### Pilot

The interview guide was piloted at the Paediatric Sickle Cell Clinic of KBTH once approval had been received from the Institutional Review Board of Korle Bu Teaching Hospital, after which modifications were made. Five participants were interviewed for the pilot study using semi-structured individual interview guides to conduct individual in-depth interviews. This provided the interviewer with a better understanding of the interview guide as well as a chance to hear some likely questions some of the interviewees had.

The interviews were transcribed, and modifications to the interview guide were made based on feedback from the pilot before the commencement of the study. Transcripts from the piloting were not added to the main interviews for analysis.

### Data collection

A semi-structured individual interview guide was developed using the conceptual framework and objectives of the study were used to conduct interviews. The inclusion criteria were explained to patients at the clinic, and parents who fit the inclusion criteria and were willing to participate were interviewed during clinic hours. The sample was heavily skewed towards mothers because purposive sampling was used, and any parent who was available at the time of interview met the eligibility criteria and consented to be interviewed. As the majority of the parents at the clinic were mothers, the sample was thus heavily skewed towards mothers. This could also be explained by the fact that in Ghana, it is mainly mothers who are primary caregivers to children; thus, they were the majority at the clinic [24,25].

### Data handling and processing

To ensure the privacy and safety of data collected, all audiotape recordings, field notes and memos were kept under lock and key and only accessible to authorized persons. Sufficient controls were put in place to preserve the safety of the data. Interviews were conducted in Twi or English and tape-recorded for transcription later. Thirteen interviews were conducted in English while eleven were conducted in Twi.

NVivo software and Microsoft Excel and Word were used to assist in the analysis and coding of data. To ensure the privacy and safety of the data collected, all audiotape recordings, field notes and memos were kept under lock and key and only accessible to authorized persons.

### Data familiarisation

The researchers familiarized themselves with the data by listening to interviews and transcribing them verbatim. Researchers also read the interview notes and the field notes. Since one of the researchers was also the interviewer for all the interviews, the researchers had a full grasp of the data and some of the expected codes. They also reviewed the audio recordings along with the written transcripts and field and interview notes to analyze initial thoughts and early patterns.

### Systematic coding and development of the codebook

Thematic Analysis following the 6 phases of thematic analysis as defined by Braun and Clark was used to establish emerging themes. Open coding using an inductive approach was used as a first step in identifying initial codes. Similar codes were then clustered to form categories, after which the relationships between the emerging codes were analyzed and categorized into sub-themes. Transcripts were loaded into NVivo, and an initial word frequency query was run by setting the minimum word length and filtering out common prepositions and verbs. This was conducted to gain insight into recurring words, and a word cloud was then created. A mind map was also created using NVivo to link codes or significant words into sub-themes and interconnecting hierarchies. Words such as sickle/sickle cell, condition, acceptance, result(s), complication, drugs (medications), hospital, and clinic pointed researchers towards emerging answers for the research questions. A line-by-line coding of transcripts was also done using Microsoft Word to identify more codes missed during the initial word frequency query. All interconnected codes were then categorized/grouped into sub-themes by analyzing the research questions and grouping recurring codes that answered each question on the interview guide. A guided list of definitions for each defined code was provided to each team member to guide them in the analysis process.

### Code refinement

The initial code generation was done by the interviewer. The data was then sent to other team members who reviewed and revised the codes. Some initial codes which were similar, such as codes under emotional responses, were merged into one, while others that were considered too broad were separated. There was also an iterative analysis of codes and sub-themes to refine them. Some codes that were part of the expectant codes list but did not generate enough repetition to appear as codes were eliminated.

### Theme development and review

Similar codes were clustered to form categories, after which the relationships between the emerged codes were analyzed and categorized into themes. Sub-themes that had the same concepts and ideas were combined to form themes. There were 7 themes and 18 sub-themes that emerged: Intrapersonal influences (Emotional Response after Disclosure, Decision to Share Results, and Adherence to Routine Clinic), Interpersonal influences (Care and Management of the Disease at Home, Knowledge of Genetics of SCD) and Structural Influences (Access to Care and Culture). Refer to the codebook in Table 1 below.

**Table 1:** Codebook.

Research Question/objective(Open coding)	Themes(Quotes from interviews)	List of Codes	Emerged Themes	Attitude(From Theoretical framework)	Construct(From SEM)
Reactions to the disclosure of positive results	*“You know, life is hard now, and I was scared that if it happens that she is a sickler, what would I do”?“Because that first time she told me, I got scared. That’s why I didn’t believe it. I was worried”.‘he still doesn’t believe. And I tell him that, if he didn’t have it, the nurses wouldn’t have told me he has it”.*	1. Scared/ Worried 2. Disbelief/Surprise/Shock	1. Emotional response after disclosure	Influences	Intrapersonal (individual)
Reactions to the disclosure of positive results	*“well, I didn’t want anyone to know. I just wanted it to be between me and my husband so I didn’t tell anyone”* *“Yes. I told the caretaker. Because if I have to leave, and you don’t give the medication, I will blame her. And then recently, I told my mother-in-law… and then my parents, my siblings are also aware”.*	1. Shared results with partner only due to fear of stigma 2. Shared results with family and friends	2. Decision to share results	Influences	Intrapersonal
Response to In-clinic counselling	*“How to how to, ehh, is it, prevent it, ehehn, in terms of not to take something cold. And all those kinds of things. You will know the way when it’s cold, you should put something, like cardigan, all those kinds of things. And you have to take your medication always, no crises, for the 0 months to 5 years. You won’t have any problem”.“When they are babies, maybe you will see one of their hands swelling or their feet swollen. Or their eyes. and when they get temperature, you have to bring them to the hospital and not stay in the house. It might be infection”.*	1. Preventive measures/care at home (nutrition, hydration, warm clothes)2. Awareness of treatment options3. Awareness of possible complications4. Danger signs	3. Care and Management of Newborns with SCD	Network (with clinic staff)	Interpersonal
Response to In-clinic education	*“Yes. She said like, it was from both my husband and I. and that I have S in my blood and my husband also has S in his blood. And that’s how come the child has the S”.“They took me through the sickling. How it forms. How their blood cells look like? Is it the white one or the, the red one? Yea, that looks like a bofloat, and theirs is like an arch, the moon, half moon, which is very hard not like the others that are soft that can pass through all the blood. Theirs because is hard, sometimes, it gets broken and chokes the veins that you have to give them the (massage) and all that. so, I was taught all that”* *“It’s me and our last born (meaning youngest sibling) who have the disease in our family.”*	1. Inheritance of SCD 2. Blood disorder 3. Family history of disease	4. Knowledge of genetics of SCD	Network with clinic staff	Interpersonal
Attitudes towards Follow -up procedures	*“I’ve been coming to clinic for the past 7 to 8 months. Every month. I’m here. Yes, sometimes even 2 weeks. In a month, I can come like 2 times”.“Because I have that experience. So I don’t want her to pass through the stress I have gone through, so I come to clinic”.“We come. Every 3 months, we come. But when he was 2 years, we were asked to come and do some labs. But we couldn’t do it. Because of the COVID. I was scared so I didn’t come.“Madam please, you see, for my son, I love him. And it’s the doctor who knows, so whatever they tell me I have to listen and do it. But sometimes, when I’m unable to come on the date, it’s usually because of financial difficulties. I may be lacking money for the labs …. But financially, it’s been difficult. Because of money issues. I was supposed to bring him last month but because I didn’t have money, I couldn’t come”.“oh it’s usually the lab we frequently do”*	1. Adherence to routine clinic visits (due to expectation of healthy outcome for child)2. Adherence due to parents’ personal experience with disease)3. Non-adherence to clinic visits due to COVID-19 4. Non-adherence due to financial difficulty. 5. routine lab tests	5. Adherence to routine clinic	Influences	Intrapersonal
Attitudes towards Follow-up Procedures	*“They checked my child and said to buy some medications. They wrote a prescription for it. And I was initially told to do the lab tests, but the paper got missing. They asked that I do the labs and bring the results. And that my insurance will cover some. they asked whether my child has been sick before? And I said no”.*	1. Cost	6. Cost	Influences	Structural
Attitudes towards Follow-up Procedures	*“uhh, you get brokenhearted, but then, God has a purpose for everybody on this earth, and Jeremiah says that ‘before I was in my mother’s womb, you knew me.’ So, umm, I know it’s the will of God. So, I have accepted that…. Uh huh, I ask God for Grace to help me take care of her”*	1. Religion	7. Culture	Influences	Structural

### Memoing and reflexivity

Sufficient controls were put in place to preserve the safety of the data as well as to guarantee its accuracy devoid of any bias. As such, the researchers used various validation strategies to ensure credibility and trustworthiness. Credibility was achieved in this study by using a codebook, which served as a trail of evidence for all data gathered and analyzed. A field diary and a memo were also used to record all observations during interviews and analysis of data. Data were triangulated using memo notes, field diaries and transcriptions from interviews in the write-up to ensure trustworthiness and credibility. Saturation was achieved in this study by utilizing the 2 principles of achieving saturation according to Francis et al [23], where the initial minimum sample size and stopping criterion were established before data collection. Another way of ensuring trustworthiness and credibility in this study was the use of 2 peer debriefers in the person of my academic supervisor, Dr. Faustina Hayford Blankson and Ms. Diana Dwuma-Badu, a Haematologist nurse specialist and a researcher at the sickle cell clinic of KBTH. Transferability, another quality of validity, was achieved by using rich, thick descriptions and quoting participants’ voices under each theme. The researcher used an iterative process of checking interpretations and analysis of data with the peer-debriefer, as well as constantly comparing the responses of participants to help influence the validity of the study.

### Ethical consideration and confidentiality

Ethical approval for this study was sought and acquired from the Scientific and Technical Committee and the Institutional Review Board of Korle Bu Teaching Hospital (KBTH-STC/IRB/00094/2021). Consent was sought from participants before interviews began. Consent forms were given to parents to read and sign, while those who could not read had the consent translated verbally into the local language for them, after which they thumb-printed their consent with a witness present. Participants’ anonymity was ensured by assigning pseudonyms to them and assuring them their real names would not be used. All information gathered was treated with the utmost confidentiality.

## Results

The themes have been presented using constructs from the Socio-Ecological Model of Health. A total of twenty-four [24] parents of children who had tested positive for SCD at birth and enrolled in the clinic were interviewed. Four of the participants had children who were less than one-year-old. Four more participants had children aged one year to two years, and sixteen had children aged two years one month to three years and eleven months. The participants consisted of seventeen [17] married and three unmarried parents. Two other participants were not married but were in a relationship with the father of their children, while two were separated from their husbands. All participants lived within the Greater Accra Region. Nine of the participants had completed tertiary education, three had completed primary six, five had completed Junior High School (JHS), six had completed vocational or Senior High School (SHS), and one had not had any formal education at all. Five of the participants had more than one child enrolled in the clinic. Ten [10] of the participants also had the disease or had partners who had the disease, while fourteen [14] participants had the SCD trait. The results are found in Table 2 below.

**Table 2:** Summary of socio-demographic information of participants.

SOCIO-DEMOGRAPHIC CHARACTERISTICS	
Baseline characteristic	Frequency(%)
***Educational level*** None/Primary Junior High/Senior High/Vocational/Technical Tertiary	4 (16.7%) 11 (45.8%) 9 (37.5%)
***Marital Status*** Married Not married/Separated	17 (70.8%) 7 (29.2%)
***Number of Children with SCD*** 1 2 or more	19 (79.2%) 5 (20.8%)
***Parent with known SCD?*** Yes (mother or Father) No	10 (41.7%) 14 (58.3%)

The codes were categorized into 3 main constructs based on the socio-ecological framework, namely: intrapersonal influences, interpersonal influences and structural influences. A summary of the codes and themes can be found in the codebook in Table 1 as follows: Intrapersonal influences (Emotional Response after Disclosure, Decision to Share Results, and Adherence to Routine Clinic), Interpersonal influences (Care and Management of the Disease at Home, Knowledge of Genetics of SCD) and Structural Influences (Access to Care and Culture). In total, there were 7 main themes and 18 sub-themes.

### Intrapersonal influences

Three themes emerged from this construct: Emotional response after disclosure, the decision to share results and adherence to routine clinic. Emotional response after disclosure had 2 sub-themes, namely: scared/worried, disbelief/surprise/shock. Under the decision to share results, there were 2 sub-themes: shared results with partner only due to fear of social exclusion and stigma and shared results with family and friends. The third theme under intrapersonal influences was adherence to routine clinic visits, and under this theme, there were 5 sub-themes: adherence due to expectations of health outcomes, adherence to routine visits due to parents’ personal experience with the disease, non-adherence due to COVID-19, non-adherence due to financial difficulties, and routine lab tests. This study examined the interplay of factors such as emotions, decisions, and knowledge and how they influence parents’ experience when receiving positive SCD results of their children. This study also examined how receiving negative news affects the emotions, decision-making, and knowledge of parents of children with SCD. Parents’ prior knowledge or lived experience elicited fear and worry after disclosure of the positive SCD results of their children.

### Emotional response after disclosure

#### Scared/Worried.

Emotions are an inherent part of individuals and are known to affect how they react to information; thus, negative news elicits negative emotions. Participants reported that they felt scared when they were initially told of the positive results. For participants, this reaction was due to the little knowledge or preconceived ideas they had about the disease.

*“It’s not easy oo. It’s not easy ((with tears in voice)). Me knowing how it is. Especially me, if I didn’t even know how it is. But me knowing how the pain, hmmm, the, the, hmm, it’s not easy oo. I’m facing a lot inside me… And I’ve been keeping this in me. And I don’t know what to do. I don’t know what to do. Hmm, it’s a really difficult thing; ever since I got to know my son has this disease.”(Female participant)*

Some participants also reported being worried after receiving the results. They reported they got worried because of the knowledge that sickle cell disease had a lot of socio-economic ramifications. As such, a positive diagnosis was very worrying for them, as it meant lots of hospital visits as well as potential financial challenges.

*“Because I can sometimes look at the child, and shake my head and think to myself, this disease too, where is it coming from”? That I gave birth and need to be at the hospital every 3 months. It’s a lot of worry.”(Female participant)*

#### Disbelief/Shock/Surprise.

Despite pre-testing counselling and post-testing counselling at the clinic, some participants reported being shocked or surprised by the results. They reported that they did not believe the results when they were initially told about them because they were not expecting the results. Some participants, however, then came to believe and accept the positive results when their children started showing symptoms of the disease.

*“And then later she called and informed us that he had the disease, but I didn’t believe it. And then when we were 10 months, he started getting sick.”(Female participant)*

*“That first day they gave me the results, I didn’t believe it.” (Female participant)*

However, some of the participants reported that even after receiving the positive results and having attended the clinic for a while, they and/or their partners still did not believe the results. This belied the parents’ understanding of the genetics and inheritance patterns of the disease.

*“No. he still doesn’t believe. He said he donates blood to people in Korle Bu all the time. So if he had the disease, they wouldn’t allow him to.”(Female Participant)*

*“So it’s only my husband and I that know. And we don’t believe in that thing, so, we don’t see our child as SC.” (Female participant)*

Some of the participants, on the other hand, had not been surprised by the positive results of their newborns as they already had prior knowledge of the family history of the disease and, as such, knew of the possibility of their baby testing positive for the disease. Some of the mothers especially knew about their carrier status due to testing during antenatal visits; thus, they were not surprised by the result.

*“I wasn’t surprised when they told me. Knowing that my other girl already had the disease. I understood.”(Female participant)*

*“So him (pointing to child with SCD at clinic), when his results came, I wasn’t surprised because I knew the dad is SC. So I wasn’t surprised at all.”(Female Participant)*

*“I wasn’t really surprised. Because I had been told that if I give birth to more children, all my children could have it.”(Female participant)*

### Decision to share results

#### Shared results with a partner only due to Fear of Stigma.

Findings showed parents’ decision to share results with a few people, including their partners. They reported that apart from their partner or the child’s father, they had not shared the results with any other person. They stated they had kept the results just between the 2 of them and not shared them with any other person due to the fear of judgment, stigma and social exclusion.

*“Well, I didn’t want anyone to know. I just wanted it to be between me and my husband, so I didn’t tell anyone.”(Female participant)*

*“Hmm, for my family, I don’t really mind telling them, but it’s because I don’t want people to say the child is this or the child is that. Yes, I don’t want someone to stand somewhere and tell another person about it and say something like oh this child is beautiful, but ahh, she has this disease. And then it will reduce her marks or something. I don’t want that. Yes, you see, people will talk. Maybe they don’t mean anything bad, but…” (Female participant)*

*“Nobody, oo. I’ve not told anybody. Because of the stigma, I don’t want to… to tell anybody… Because of what I went through, I don’t want to tell people about it. My mom didn’t keep quiet, and people would tease me and all that. So, I don’t want that.”(Female participant)*

#### Shared results with family and friends.

Some parents, however, did not mind sharing the results with some family and friends as they felt those people were closer to them and were part of providing care for the children. As such, they felt they needed to know to help them care for the child.

*“Yes. I told the caretaker. Because if I have to leave, and you don’t give the medication, I will blame her. And then recently, I told my mother-in-law… and then my parents. My siblings are also aware. So that, in case there is something to give, I tell you, chilled things are not for her. I’ll tell you, please, hold on.”(Female participant)*

### Adherence to routine follow-up procedures

#### Adherence due to the expectation for a healthy outcome for the child.

Parents reported attending the clinic regularly because they wanted their children to remain healthy; thus, they came in every time they were scheduled for clinic every few months. They also mentioned the physical examination that is done on their children to ensure they were healthy.

*“I’ve been coming to the clinic for the past 7 to 8 months. Every month. I’m here. Yes, sometimes even 2 weeks. In a month, I can come like 2 times … for me, I always say that because of his health. I want him to be healthy. And for his breathing to be fine. Because sometimes, when we come, they check everything- his breathing- and they do a physical exam of him to make sure everything is fine. So, I bring him to make sure everything is fine. Because when we are home, everything is fine. But when I’m given a date to come, I have to come.”(Female Participant)*

#### Adherence due to parents’ personal experience with the disease.

Parents reported adhering to regular monthly or bi-monthly clinic visitation due to their own experience living with the disease and, as such, found it necessary to keep their child healthy. Lived experiences of the parents thus were a contributing factor to adherence to follow-up clinical procedures, with many reluctant for their children to experience ill health and hospitalisation as they did.

*“Because I have that experience. So I don’t want her to pass through the stress I have gone through, so I come to the clinic.”(Female participant)*

#### Non-adherence due to financial difficulties.

There were, however, some who reported that they sometimes missed appointments due to circumstances such as financial difficulty.

*“Madam, please, you see, for my son, I love him. And it’s the doctor who knows, so whatever they tell me I have to listen and do it. But sometimes, when I’m unable to come on the date, it’s usually because of financial difficulties. I may be lacking money for the labs …. But financially, it’s been difficult. Because of money issues. I was supposed to bring him last month, but because I didn’t have money, I couldn’t come.”(Female participant)*

#### Non-adherence due to the COVID-19 pandemic.

Some of them also reported that they did not attend the clinic for a while due to the COVID-19 pandemic. They reported that even though they were scheduled for regular clinic visits, some of these circumstances prevented them from visiting the clinic regularly.

*“We come. Every 3 months, we come. But when he was 2 years old, we were asked to come and do some labs. But we couldn’t do it. Because of COVID. I was scared, so I didn’t come. And then I was called in November to bring her. And we will come again in February.” (Female participant)*

### Routine laboratory tests

Parents reported that they were routinely asked to do lab tests such as full blood count, reticulocyte count, and sometimes liver function tests, among others. This, they stated, was done routinely, most often once a month. Participants reported that they usually adhered to whatever lab requests they were given to do.

*“And if I have to do any labs, if I have money, I hurry and do it, so the doctors can check his blood levels. So, I believe what they say is good for me.” (Female participant)*

*“Hmm, so far, the labs are the issue. Most times, you have to do labs every month and send them to the clinic. And their medication. And their feeding and nutrition. It all costs”. (Female Participant)*

### Interpersonal influences

Themes under this construct emerged after looking at the network /relationship between parents and clinic staff and the counselling given to them. They reported that they had been educated on the care and management of their children with SCD at home, as well as the genetics of SCD, during counselling and educational sessions as part of comprehensive follow-up care provided at the clinic. Two themes emerged from this: Care and management of newborns with SCD and knowledge of the genetics of SCD.

Under care and management of newborns with SCD, 4 sub-themes emerged, namely: preventive measures/care at home (nutrition, hydration, warm clothes), awareness of treatment options, awareness of possible complications and danger signs. There were 3 sub-themes which emerged under knowledge of the genetics of SCD, and they were: inheritance of SCD, blood disorder and family history of the disease. Interpersonal factors such as counselling and education influenced parents’ adherence behaviors to clinical care.

## Care and management of children with SCD

### Preventive measures

Parents reported they were counselled on how to care for their children at home and how to take preventive measures to reduce illnesses and painful episodes at home.

They reported that they were counselled on how the nutrition or diet of the child is important and could improve the health of the child.

*“They said to pay attention and take care of him well, his eating habits. The food he eats... They said I should pay attention to that. So that he doesn’t become sick. Yes.”(Female participant)*

Some parents also stated that they were advised to make sure the child drank lots of water and was always hydrated. Thus, they were advised on the importance of hydration for their children with SCD.

*“Yes. Hmm, like coming to clinic, they will take her Hb. One, they will check her Hb, and 2 they will make sure you give him/her some medication. So that you will not have crises. Give a lot of water. Fruits and mind the kind of foods he/she eats. Yes, so coming to clinic helps me a lot” (Female participant)*

Parents reported that they were also educated on the need to keep their children warm and to prevent them from extreme temperatures.

*“Hmm, they said many things. How to, how to, eh, is it, prevent it, ehehn, in terms of not to take something cold. And all those kinds of things. You will know the way: when it’s cold, you should put something on, like a cardigan, all those kinds of things. And you have to take your medication always, no crises, for 0 months to 5 years. You won’t have any problem.”(Female participant)*

### Awareness of treatment options

Medication literacy was one of the things imparted to parents as well, and as such, parents reported that they were now aware of the medication as well as other treatment options necessary for managing the disease. Parents reported being advised to give their children penicillin, zinc supplements, folate supplements and hydroxyurea.

*“He’s taking, umm, brufen, zincovit, folic acid, penicillin V, but recently he was given the … I’ve forgotten the name… yes. Hydroxyurea” (Male and female participants)*

*“And the doctor said not to think too much of it. So, when he keeps taking the medicine, small, small it will become fine. Maybe the SS too can change. Because it’s good to treat them when they are little. Before they grow up.”(Female participant)*

### Awareness of possible complications from the disease

Parents stated that they were counselled on the possible complications that could emerge from the disease. They reported that they were taught about how the disease could affect other parts of the body such as the eyes, the kidneys, etc.

*“They told me the disease can affect other parts of the body. That’s why I was so sad when I couldn’t get the eye test for my son when his eye got swollen.”(Female participant)*

### Awareness of danger signs

Parents informed us they were counselled on what danger signs to watch out for and actions to take to mitigate any

symptoms at home. Parents were counselled and gained knowledge on watching out for danger signs such as yellow eyes, fever, swollen feet and hands, constant crying, etc.

*“They said he may get complications from the disease. When they are babies, maybe you will see one of their hands swelling or their feet swollen. Or their eyes. And when they get a temperature, you have to bring them to the hospital and not stay in the house. It might be an infection.”(Female participant)*

## Knowledge of the genetics of SCD

### Inheritance of SCD

They reported that they were taught that the disease was inherited from both parents. Parents were made aware of the fact that they both had to have the trait to be able to have a child with the disease and that the disease was found in their genes and was passed down to their child. They also reported being informed of the possibility of having other children with the disease.

*“Yes. The day I brought the results, the doctor asked that both my husband and I come. So, we came, and he explained everything to us. They explained everything. That because my husband is AS and I’m AC, that’s how come. He took my husband’s S and took my C. The older one took my husband’s S and my A.”(Female participant)*

*“They told us. Ehh. They told us that it’s genetic. If one of the parents has, then probably 1 or 2 of your children will have it.”(Female participant)*

### Blood disorder

Parents were educated on the genetic makeup of sickle cell disease, as well as its inheritance patterns. The fact that sickle cell disease is a blood disorder and that the blood cells are sickle-shaped instead of round and break easily, thus causing painful episodes, was made clear to parents. Painful episodes are caused by blockage of blood vessels by the deoxygenated sickled cells, and this was explained to all parents during genetic counselling in clinic.

*“They took me through the sickling. How it forms. How their blood cells look like. Is it the white one or the, the red one? Yeah, that looks like a bofloat, and theirs is like an arch, the moon, half-moon, which is very hard, not like the others that are soft that can pass through all the blood. Theirs, because it is hard, sometimes it gets broken and chokes the veins that you have to give them the (massage) and all that. So, I was taught all that.” (Female participant)*

*“They told me that there is S in the child’s blood. And she showed me how it is, with a diagram, how the S looks in the blood, she drew it and showed me. And how that, for us, the blood is not round but is curved. And that is how come it happens that way. And so, they will try and treat it. She said it is something that’s happening in Africa. Sickle cell disease. And she said so nowadays when they see that a child has the disease, they advise parents to bring the child to the hospital for care so that it can prevent certain complications.”(Female participant)*

### Family history of the disease

As part of in-clinic counselling, participants also stated that they were asked if they were aware of any known family member with the disease. They reported that they were informed that sickle cell was a familial disease passed down from parents to the child and could affect other family members.

*“Because my father, for instance, he also has it … we are 3. My sister also has the disease. Hers is worse.”(Female participant)*

*“It’s me and our last born (meaning youngest sibling) who have the disease in our family.”(Female participant)*

Despite pre-screening counselling and in-clinic counselling, some participants had misconceptions about the inheritance and genetics of the disease, and this thus affected their reactions to the disclosure of positive results. This is further explored in the discussion section.

### Structural influences

Structural factors in this study included access to health facilities and culture. The study examined how factors such as access and culture influenced parents’ experience of receiving positive SCD results for their children. The interplay of factors such as economic constraints and culture’s influence on parents’ reaction to receiving SCD results, their decision to share these results and adherence to routine clinical follow-up processes were also explored.

### Cost

Cost as it relates to health in this study refers to the cost of medication, lab tests, as well as transportation to and from the clinic during scheduled visits, as well as other costs incurred during hospitalizations and hospital visits. The cost of management of SCD is known to be very high in certain jurisdictions and can be a barrier to accessing clinical care.

In this study, participants stated that the country’s National Health Insurance Scheme (NHIS) covers the costs for some of the medications (zincovit, folic acid and hydroxyurea) as well as the costs of some routine lab tests such as full blood count and reticulocyte count. Other tests and medications are covered out of pocket, with parents having to find ways to offset these costs.

*“They checked my child and said to buy some medications. They wrote a prescription for it. And I was initially told to do the lab tests, but the paper got missing. They asked that I do the labs and bring the results. And that my insurance will cover some. They asked whether my child has been sick before? And I said no.”(Female Participant)*

*“So, he hasn’t made up his mind to look for a job to get money to help with the labs. But. I’m the one forcing him to get money for it [for him]”(Female Participant)*

### Culture

Culture plays a significant role in how people interpret information as well as people’s knowledge of phenomena. Cultural factors such as religious beliefs, gender norms and expectations have implications for health-seeking behaviour.

### Religion

In this study, the role of religion was evident, with parents expressing their religious beliefs about the disease, with many believing in a spiritual being to help cure their children of the disease or providing a way out financially.

*“Uhh, you get brokenhearted, but then, God has a purpose for everybody on this earth, and Jeremiah says that ‘before I was in my mother’s womb, you knew me’. So, umm, I know it’s the will of God. So, I have accepted that…. Uh huh, I ask God for Grace to help me take care of her”(Female Participant)*

Religion plays a huge role in Ghanaian culture, and there was mention of how the various religious institutions, such as the churches, play a role in promoting genetic testing through pre-marital counselling provided by these churches.

*“Yes, the education. I know a lot of the churches do that. They ask the couples to check” (Female Participant)*

## Discussion

### Objective 1 – Reactions of parents to disclosure of initial results

The first objective of this study was to explore how parents reacted when the initial positive results were disclosed to them. After data analysis, two themes emerged that provide an answer to this question: emotional response to the disclosure of results and the decision to share results.

Parents reported feeling scared, worried, in disbelief and surprised due to all the negative information they had heard about the disease. As such, they felt scared and worried that their children could also suffer from the disease. This doesn’t differ much from a study by Chudleigh et al [26] where they explored the experiences of parents of newborns receiving positive SCD carrier results. In that study, parents reported a range of emotions such as devastation, guilt, denial, surprise and shock. As much as these emotions may not have been the same, they were still negative emotions that were felt by parents receiving positive SCD results about their newborns. One notable finding from this study was that, despite being aware of their own carrier status or the prior diagnosis of their older children with the condition, several parents showed surprise or shock at their children’s results. One explanation for this could be that, while being aware of their own status, some of these parents lacked the knowledge necessary to recognize that their children might have the illness. These various emotional reactions shed light on how this may impact the initial acceptance or rejection of positive results, subsequent clinic attendance, medication adherence, and routine laboratory testing. It would be expected that parents who exhibit shock or surprise would not be more inclined to accept positive results, which could subsequently have an impact on their adherence and subsequent clinical attendance. The opposite would be true for parents who did not express shock or surprise.

Prior knowledge of the disease, as well as the possibility of having a child with SCD, could be argued to have an impact on the reactions of parents to receiving positive SCD results for their children, and this could then affect clinic attendance and adherence to medication and routine lab tests. Parents who knew about the disease were more accepting of the positive result and more willing to heed counselling recommendations and were also more likely to attend scheduled routine appointments. Conversely, parents with little or no prior knowledge were less inclined to accept the results, particularly when their children were below six months of age and showed no symptoms of the disease. This study therefore reiterated the importance of educational awareness of SCD in addition to pre-marital counselling and SCD screening. This also has significant importance in clinical settings because prior knowledge was known to affect how parents reacted to counselling and education on the disease. Prior knowledge should therefore be taken into account when designing SOPS and guidelines for SCD counselling in clinical settings.

Another significant finding from this study was that parents who either had the disease themselves, had a family member who had it or had another child who had the disease were less surprised and therefore more accepting of the initial positive results whiles parents who only knew about the disease but had no real experience with it were still surprised about the results. This indicates the importance of lived experience and how it has more of an impact on parents’ emotional responses to initial positive results. It could also be argued that prior knowledge alone is not enough, and therefore antenatal SCD testing for parents during pregnancy and genetic counselling could have a greater impact. Another significance of lived experiences of parents was that parents were more likely to adhere to follow up/routine visits since they had first-hand knowledge of the devastating impact of the disease and did not want the same for their little children. Prior knowledge of SCD, especially the negative views, could lead to doubt when parents are given a positive diagnosis [27]. In this study, parents’ reasons for disbelief were due to misconceptions and misinformation about the disease and, as such, they believed that their children could not have the disease since their newborns looked very healthy at the time.

Parents who had prior knowledge due to a family history of the disease stated that they were not surprised by the results, while those who may have heard about the disease but did not know their carrier status were scared, worried, and surprised by the results for their children. Thus, prior knowledge of the disease impacts parents’ emotional reactions to the disclosure of positive SCD results of their children.

The decision to share the initial positive results was influenced by fear of discrimination and social exclusion due to the sometimes physical effects that the disease could have. In this study, most parents of children with SCD decided to share the positive results/ or diagnosis with someone, at least a partner, with some preferring to keep the diagnosis just between them and their partners. Some parents’ decision not to share the results was based on the desire not to subject their child to stigma and also to prevent their child from being less socially accepted among friends and even family.

This is in contrast with the study by Ulph et al [28] documenting parents’ responses to receiving sickle cell carrier results for their children, where parents felt a responsibility towards sharing the results with their extended family, and parents had no struggle with informing extended families about the results. For others, the decision to share with at least just their partners was because of the expected support they could receive from them. The study by Chudleigh et al [26] also contrasts the findings in this study, as that study reported that most of the parents of newborns who had received positive SCD carrier results for their newborns shared results with family and friends. In this same study, however, some of the parents who had lived in Africa or had family back in Africa hesitated to inform that side of their family in Africa because they felt their families had a bleak outlook on the disease. It can therefore be said that geographical context and culture influence people’s experience of stigma, and this in turn may influence parents’ decision to inform other people about the results of their children.

Parents’ decisions to share or not share their children’s positive SCD findings were therefore heavily influenced by social acceptance and stigma; many parents chose not to share because they did not want their children to face discrimination. The important thing to note, though, is that stigma, social exclusion, and prejudice continued to be a problem for all parents, even those who chose to share their children’s positive SCD results with friends and relatives. They made the decision to share even though they were aware of the potential harm sharing would do to their kids. This is particularly important for instructors when creating education and learning materials. Stigma and social exclusion were also the reasons why some of the parents expressed shock or worry when they received positive results because they knew enough about the diseases to know how stigmatizing it was. Stigma is therefore a huge factor for SCD newborn screening, and should be factored in when designing pre-SCD screening counselling materials as well as post -screening materials. In clinical settings, it is significant that parents are educated on the genetics of the disease so as to reduce blame. It is also important that stigma-free spaces are provided in clinical settings to help provide privacy and reduce stigma.

Another significant contribution of this study was that those who had lived experience of the impact of the disease were less likely to share the results even with their family members. They attributed this to the lived experience of the stigma and social exclusion that they themselves faced because their parents had shared their own positive results. On the other hand, those who either had no prior knowledge of the disease or had prior knowledge but no lived experience with the stigma attached to the disease were more likely to share the results, with the focus more on ensuring enough care for their children rather than focusing on the stigma. It could therefore be summarized that lived experience of the disease had a greater impact on emotional response as well as the decision to share results. Social acceptance and stigma were thus a major part of the decision of parents to either share or not share the positive SCD results of their children.

### Objective 2- In-clinic counselling and comprehensive follow-up care

The second research question aimed to look at how parents responded to in-clinic counselling and education and how this formed an essential part of comprehensive follow-up care. It aimed to provide insight into parents’ relationships and interactions with staff at the clinic in terms of the counselling and education provided by trained staff at the clinic. Analysis of data yielded two themes that answered this question: care and management of children with SCD and knowledge of the genetics of SCD.

Counselling patients with sickle cell disease is an essential tool in ensuring effective adjustment [27]. Educating parents on how to care for their children at home is one of the ways of reducing bacterial infections in children [29,30]. Parents should be advised to give their children enough fluids to reduce dehydration. They should also be advised to wear warm clothes in cold weather and the need to ensure proper diet and nutrition [29,31,32].

In this study, parents of children were counselled in clinic and received a lot of knowledge on the nature of sickle cell disease, preventive measures and caring for their children at home. Parents were advised during counselling sessions with staff to make sure to take care of their children at home by ensuring they stayed hydrated, warm and had adequate nutrition, as well as watching for danger signs such as yellow eyes, coke-colored urine, bloated stomach, swollen joints, and excessive crying. Parents were also advised on the treatment options available for their children. As such, parents knew what medication they were prescribed and how that medication played a role in managing the disease. This is consistent with a study by Colah et al [33] documenting the Indian experience of newborn screening for SCD, where parents were given daily folic acid and penicillin prophylaxis for their newborns. It is also consistent with the Jamaican experience study by King et al [34], where parents of newborns diagnosed via newborn screening were counselled on the need for routine penicillin prophylaxis. Medications such as folic acid, vitamin C, and penicillin have been known to reduce infections among children with SCD.

Knowledge of the genetic make-up and inheritance pattern of the disease is known to reduce misunderstandings within families, as women alone will not be blamed for giving birth to children with SCD. Knowledge of danger signs as well as how to care for children, as demonstrated in this study, helps parents make informed decisions on how to care for their children and when to seek medical help. SCD in children under five years is known to cause complications. Complications such as vaso-occlusive pain episodes are known to be the most common clinical manifestation of SCD, and teaching parents to spot these signs early and seek help at health centres has been known to prevent worsening episodes and even death. Acute chest syndrome, one of the leading causes of morbidity among children with SCD under 5 years of age, is known to require early intervention, and parents being aware of the early signs plays an important role in seeking early intervention for their children at emergency rooms of health facilities. Parents being aware of symptoms and danger signs also gives them a huge advantage to intervene in the treatment of their children in clinic and during emergencies or admissions. Counselling of parents on danger signs and symptoms of SCD in their children is therefore an important component of care, as parents are usually the first assessors of any illnesses among children, and early identification means early seeking of health attention and early intervention, thus saving lives. Educating parents or caregivers on the care and management of SCD is thus a way of empowering them. Once a sickle cell diagnosis has been confirmed after the newborn screening, parents need to be educated on the type of SCD their child has, the genetics of the disease and how to manage the disease at home. Educating parents on the genetics of sickle cell disease is essential, as illustrated by this study. Parents were educated on what the disease was, its causes, how the disease is inherited and how family history plays a role in the inheritance of the disease. This study showed that as part of comprehensive follow-up care for newborns diagnosed with SCD at birth, parents need to be taken through several counselling and educational sessions or programs with trained SCD nurses or staff trained in genetic counselling. This is to provide information about the genetic makeup of the disease, the way the disease is inherited through the genes of biological parents and how the history of the disease in the family may play a role in families having a better understanding of the disease makeup. Knowledge of the genetics of SCD does have a significant impact on families’ decision-making on future pregnancies. It also has an impact on whether parents of children with SCD accept positive results of any subsequent SCD test for any future children they may have. Knowledge of the disease also plays an important role in empowering parents to make decisions to get any other children they have tested and even getting other family members tested. Another significance for parents knowing the genetic makeup of the disease is that it could help them better understand and finally accept the positive results of their children. Counselling pre- and post-newborn screening is therefore an important vehicle to promote educational awareness of the inheritance patterns of the disease. A study by Ajinkpang et al in 2022 [35] on the knowledge and home-based strategies among caregivers of children with SCD in Ghana found that even though the majority of the caregivers were aware of the type of SCD of their children, knowledge on the genetic cause of the disease was lacking. In countries such as Ghana, where over 70% of the population identify as Christians, there have been many misconceptions and myths about sickle cell disease, with some believing that SCD is a curse or that children with SCD are evil [36]. Counselling parents on the genetics of SCD would go a long way towards undoing these misconceptions by providing a scientific explanation for the disease makeup and physical manifestations of the disease. A study by Dennis-Antwi et al in 2011 on the lay perceptions of sickle cell disease in Kumasi, Ghana found that spiritual beliefs had a significant role in how parents described and understood the disease [37].

It is important to note that the counselling provided before screening and after screening in the clinic did not provide enough information to parents on how to navigate the issue of stigma within their communities. Parents also did not seem to be provided with enough resources on how to handle stigma. Even though parents had access to clinical psychologists, these psychologists were not readily available at the clinic venue, and parents had to specifically request a psychologist instead of being offered one and had to be directed to the Psychology Unit to schedule an appointment with a psychologist. This does have significance for clinical care pathways. Another important gap was the fact that even though parents knew about what medication to take and what lab tests to do, and how to navigate that whole process, they did not have enough education or awareness of the impact of the medication on disease modification as well as its implications for future health journeys of their children. This is similar to a previous study by Kai et al [38] on communication of carrier status of newborn screening results to parents, where parents reported being poorly prepared to receive positive results due to inadequate information provided during pre-screening or being overloaded with information during antenatal visits before screening. Future clinical counselling should therefore include a more proactive approach to providing psychological support in clinic, not just to the parents but to the children as well. More effort is also needed to design counselling programs that offer more holistic information on the disease-modification impact of the medication.

### Objective 3- Parents’ adherence to follow-up care procedures

The third research question sought to explore parents’ attitudes towards follow-up care procedures, i.e., adherence or non-adherence. One major theme emerged from this section, namely adherence to routine clinic care. Routine clinic visitation, regular physical examinations and recording of clinical and laboratory information of patients with SCD are important [29,39]. Routine clinical procedures for parents at the Korle Bu Teaching Hospital included routine lab tests, daily medication and routine clinic visits for physical examinations. The first five years of life of a child with SCD are very delicate, and adherence to routine clinical care is one of the sure ways to reduce morbidity and mortality of children under five years [13,29]. Up-to-date vaccinations help reduce infections and sepsis, which are known to be a leading cause of mortality for children with SCD. Adherence to taking vitamin C, Folic acid, and penicillin every day is known as a sure guide to help prevent infections and complications of the disease whiles routine lab tests such as full blood count and reticulocyte count are known to help detect haemoglobin levels and infection risks. It is therefore important for parents to adhere to routine visits every two to three months. Parents adhered to these routine clinic requirements due to their desire to see their children healthy, as well as from their own experiences with the disease. This is in contrast with a study by Ingerski et al [40] on clinic attendance of youth with SCD on Hydroxyurea, where it was found that family size, financial capacity, incidence, and financial difficulties were some of the reasons why parents could not adhere to the routine clinic visitation and lab tests, as they could not afford them.

Sickle cell disease is known to have a huge socio-economic significance for families, and in Ghana, people have described the disease as a large evil pot consuming any form of wealth within any family that has a child with the disease [36,37]. Cultural interpretations of the disease among different ethnic groups in Ghana, therefore, all contributed to parents’ negative perceptions of the disease. Structural factors such as access to health care (distance from hospital, household income, cost, etc.) also play an important role in determining and influencing adherence to routine clinical care. In this study, all the participants did indeed live within Accra; it is still important to note that distance from a health facility influences access to health care, with those closer to the health centre more likely to attend clinic when scheduled.

In this study, some of the routine lab tests and medications were covered by Ghana’s National Health Insurance Scheme (NHIS) while others were not. Money was therefore a huge factor in when parents would go to the clinic or do lab tests or buy medications for their children, with some stating that they did not attend the clinic whenever they did not have money. Factors such as access and cost were therefore seen as very important barriers to health for mothers of children with sickle cell disease. Culture also played a key role in influencing parents’ emotional response to positive results, decision to share results as well as adherence. The influence of how sickle cell disease is perceived in Ghanaian culture meant that parents who had prior knowledge of the disease knew of the negative connotations attached to the disease. Some parents mentioned having to sneak off at dawn to leave their houses to attend clinic with their children to prevent their neighbors’ from seeing them. This also boils down to the stigma attached to the disease. These are very important factors to take into consideration when designing public awareness or educational messages by factoring in ways to demystify the myths around the disease. Another important cultural aspect was the role of religious bodies and entities in creating awareness of the disease as well as providing pre-marriage counselling and testing. This is also another area where more resources are needed for collaboration between these religious bodies and health institutions. Religious institutions in Ghana play an important role by providing pre-marriage counselling and screening for couples’ eager to marry, and people tend to listen to their pastors and other religious leaders more than their doctors and nurses. As such, a consensus needs to be reached by bringing the religious leaders to the fold and training them on SCD and equipping them with the correct information to give to their members.

### Limitations of the study

One limitation of the study is that this study did not explore the role of psychosocial factors in influencing parents’ acceptance of positive results and their attitudes toward routine follow-up care.

Another limitation of this study is that the opinions of the clinic staff and other people involved in the provision of comprehensive care were not included. This may have given a more thorough insight into the experiences of parents of children at the clinic, especially concerning the counselling and education offered to parents.

## Conclusions

This study reiterates the point that parents play an important role in providing comprehensive follow-up care for children who have tested positive for SCD. Parents’ reactions to disclosure of positive NBS results, as well as their acceptance of the results, were influenced by their prior knowledge and/or experience of the disease, as well as the fear of stigma. This calls for more community-centered engagement and education to create awareness about the disease. There is also a need to strengthen measures which provide psychological and socio-economic support to families attending the KBTH clinic. There is also a need to promote a community-based, family-led model for SCD care through training of primary healthcare workers, which could in turn reduce the geographical and financial barriers to health access.

### What is already known on this topic

1. Early identification of newborns with SCD and the provision of follow-up care help reduce under 5 morbidity and mortality.
2. Parents report diverse feelings when given positive SCD results for their newborns.

### What this study adds

1. Prior knowledge of sickle cell disease impacts parents’ reactions to the initial disclosure of positive SCD results of their newborns.
2. Parents play a vital role in providing comprehensive follow-up care for newborns diagnosed with SCD at birth.

## Supporting information

S1 Table Transcripts of interviews with parents of positive newborns of sickle cell disease. (XLSX)
